# Binocular Indirect Ophthalmoscopy Complements Non-contact Wide-field Imaging with Optos to Treat a Baby Outside ETROP Guidelines

**DOI:** 10.4274/tjo.90699

**Published:** 2018-10-31

**Authors:** Özdemir Özdemir, Chetan Kantibhai Patel

**Affiliations:** 1 Oxford University Hospitals, John Radcliffe Hospital, Oxford Eye Hospital, Paediatric Vitreoretinal Service, Oxford, United Kingdom

**Keywords:** Binocular indirect ophthalmoscope, Optomap, Optos, retinopathy of prematurity, ultra wide-field imaging

## Abstract

We report a male premature baby who was born at 24 weeks gestation weighing 600 grams. There was bilateral stage 2, zone 2 retinopathy of prematurity (ROP) without plus disease at 38 weeks postmenstrual age. Ultra-wide-field Optomap images obtained 1 week later showed no change in ROP stage. At 40 weeks postmenstrual age, stage 3, zone 2 ROP was detected using binocular indirect ophthalmoscopy and documented using Optos. Minor tortuosity and dilation of vessels was interpreted as pre-plus disease. One week later, at 41 weeks postmenstrual age, Optomap images identified progressive extraretinal fibroproliferation in the nasal quadrant. As a result, the baby was treated with fundus laser photocoagulation.

## Introduction

Retinopathy of prematurity (ROP) was first described by Terry^1^ in 1942 under the name retrolental fibroplasia. ROP occurs because the retinal vessels and neural retina of a preterm newborn are incompletely developed at birth and do not grow normally. It is one of the leading preventable causes of childhood blindness, especially in high- and middle-income countries.^2,3,4^

At the present time, ROP screening is generally performed by binocular indirect ophthalmoscopy and/or wide-field digital imaging systems. There are 2 kinds of wide-field viewing systems in use for the pediatric age group: contact (3 nethra Neo, ICON, PanoCam, RetCam) and non-contact systems.^5^ The Optos uses a non-contact ultra-wide-field dual wavelength laser camera that is able to capture high-quality images from infants with ROP.^6^ The Optomap is a panoramic digital image generated by Optos scanning laser technology which shows approximately 82% of the retina.

In this report, we present a case of ROP managed using the Optos ultra-wide-field imaging system.

## Case Report

A male baby was born at 24 weeks of gestation and birth weight of 600 g. Maternal history included no clear reason for prematurity such as preeclampsia or maternal chorioamnionitis. The baby needed oxygen therapy in the first weeks of life due to chronic lung disease. 

**Figure 1:**
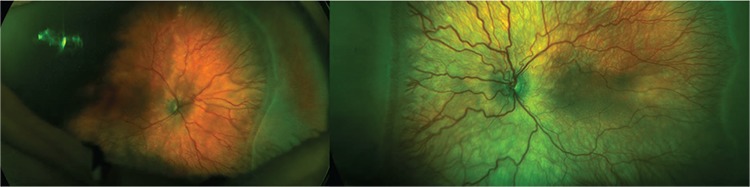
Pseudo-color fundus images obtained with Optos show bilateral extraretinal fibroproliferation in the nasal quadrant. Mild pre-plus disease is more significant in the left eye

**Figure 2:**
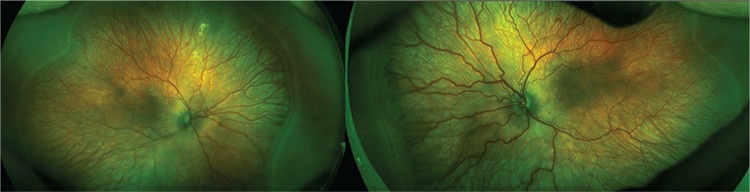
The Optomap images demonstrate stage 3 retinopathy of prematurity in zone 2 with pre-plus disease and extraretinal fibroproliferation in the nasal quadrant. There are ridge formations in the other quadrants

ROP screening was started at 31 weeks postmenstrual age and no ROP was detected. The infant developed stage 2, zone 2 ROP without plus disease at 38 weeks postmenstrual age, and was followed at 1-week intervals. There was no change in terms of ROP at 39 weeks postmenstrual age. In follow-up examination at 40 weeks postmenstrual age, the patient exhibited stage 3, zone 2 ROP with pre-plus disease. Optomap images showed pre-plus disease with minor tortuosity and dilation of the vessels (Figure 1). One week later, an area of stage 3 ROP covering 3 clock hours was seen in zone 2 with pre-plus disease. Optomap images produced by the Optos^®^ (Optos PLC, Dunfermline, Scotland, United Kingdom) imaging system demonstrated progression of the extraretinal fibroproliferation in the nasal quadrant. Binocular indirect ophthalmoscopy supported the decision to treat with laser therapy, as the neovascular areas were beginning to exert traction on the vitreous, suggesting a high risk of retinal detachment (Figure 2).

**Figure 3:**
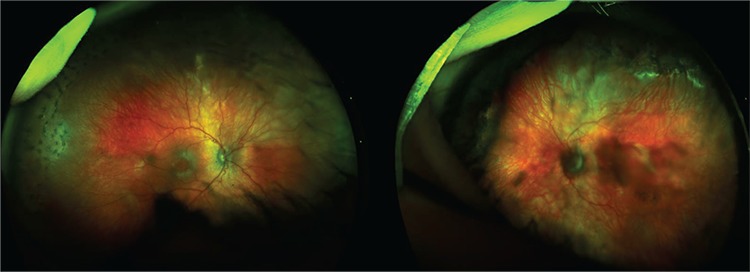
The Optomap images of both eyes show resolved stage 3 retinopathy of prematurity and regressed plus disease after argon laser photocoagulation

He was treated with fundus photocoagulation using a 514 nm wavelength argon laser. A total of 1320 laser spots were applied to the right eye and 1490 laser spots to the left eye, ranging from 140 to 200 milliwatts. There were no complications after laser treatment. Three weeks after argon laser therapy, the ROP and plus disease were fully resolved. Non-contact ultra-wide-field imaging confirmed retinal attachment with no vascular tortuosity or enlargement (Figure 3).

This case has been reported in accordance with the ethical principles in the Declaration of Helsinki and written informed consent for publication was received from the patient’s parents.

## Discussion

Ultra-wide-field imaging systems have brought about improvements in the management of ROP. They are useful for visualizing and documenting retinal features and determining ROP type.^5^ The Optos ultra-wide-field imaging system is able to obtain clinically useful high-quality fundus images from ROP patients. The Optos scanning laser ophthalmoscope can be used to demonstrate ROP and plus disease, influence treatment decisions and timing, and document resolution postoperatively.^6^ Non-contact scanning laser fundus imaging has become widely available globally in the management of adult ocular conditions such as diabetic retinopathy and retinal vascular occlusions.^7^ The Optos system is a confocal scanning laser ophthalmoscope that uses the optics of an ellipsoid mirror to capture images of the retinal periphery. The Optomap delivers a detailed 200º image of the retina in less than half a second without the use of mydriatic agents.^8^

In our patient, stage 3 ROP was detected nasally in zone 2 with pre-plus disease using a binocular indirect ophthalmoscope at postmenstrual age of 41 weeks. We were able to image the posterior pole and retina periphery with Optomap images, even the avascular area anterior to the ROP line. The Optomap images showed the extraretinal fibroproliferation at the nasal quadrant could present a risk for future detachment or dragging. The high-resolution retinal images provided by this system gave us the opportunity to discuss how to manage the case. Even though the infant did not develop plus disease and was out of the Early Treatment for Retinopathy of Prematurity Study criteria, we decided to treat the ROP disease based on demonstrating progression of stage 3 ROP. An important point is that although the Optomap provides high-quality images, they are 2-dimensional. In contrast, an indirect ophthalmoscope enables 3-dimensional visualization of the fundus and ROP features.

Retinal imaging in patients with ROP using ultra-wide-field imaging system has recently been popularized by Patel et al.^9^ They were the first team to report that the Optos was capable of capturing high-quality images in infants with ROP. Patel et al.^9^ showed that a large view of the infant’s retina can be obtained with the Optos non-contact ultra-wide-field fundus imaging system. They demonstrated the different stages of ROP at the posterior pole and peripheral retina with Optos scanning laser ophthalmoscope using a modified “flying baby position”. One of the important results from their study is that the Optomap can identify “skip areas” missed by initial laser treatment in the peripheral retina.^9^ In the current case, we analyzed the patient’s retina and the retinal periphery after laser photocoagulation and found no missed areas.

The non-contact high-resolution ultra-wide-field system has other advantages for pediatricians, neonatologists, and ophthalmologists in the evaluation of premature or full-term babies. Yusuf et al.^10^ used Optomap images to show the retinal features of 10 eyes of 5 consecutive infants (aged 1-15 months) with suspected abusive head trauma. It is indisputable that to document and record any abnormalities in patients is a medicolegal obligation. It is also possible to acquire fluorescein angiograms in premature infants with ROP using the Optos system. Fung et al.^11^ reported the fluorescein angiograms of 3 premature infants with ROP using oral fluorescein, at a dose of 25 mg/kg of body weight. The 2% fluorescein was mixed with infant formula milk and/or bottle-fed to the infants 30 minutes before the imaging process.

Although indirect ophthalmoscopy is still the gold standard method for ROP screening, non-contact ultra-wide-field fundus imaging offers new opportunities in the evaluation of pediatric retinal diseases. These 2 methods can be used together in the evaluation of ROP. In the present case, high-quality Optomap retinal images assisted us in effectively managing ROP.
